# Pediatric Tracheotomy: Comparison of surgical technique with early and late complications in 273 cases

**DOI:** 10.12669/pjms.35.1.132

**Published:** 2019

**Authors:** Murat Gumussoy

**Affiliations:** 1 Murat Gumussoy, M.D. Assistant Professor, Otorhinolaryngologist, Department of Otolaryngology Head and Neck Surgery, University of Health Sciences, Izmir Tepecik Training and Research Hospital, Izmir, Turkey

**Keywords:** Complications, Indications, Pediatric tracheotomy, Surgical technique

## Abstract

**Objectives:**

This study was aimed to compare the early and late complications of tracheotomy in pediatric patient, with respect to surgical techniques.

**Methods:**

The relationship between demographic characteristics, surgical techniques obtained from the files of the children and complications developing after surgery were compared retrospectively.

**Results:**

One hundred fifty two out of 273 developed complications after tracheotomy. Among these, 75 were early complications and 77 were late complications. Results obtained concerning early complications showed a significant difference between Skin incision and Bleeding and Accidental decannulation; Tracheal incision and Subcutaneous emphysema; surgical time and accidental decannulation and tube/ventilation problem; Surgeon’s skill level and bleeding. As regards late complications there was a significant difference between Intubation Time and Stomal-tracheal granulation; Tracheal incision and Stomal infection; Surgeon’s skill level and Stomal-tracheal granulation.

**Conclusions:**

In pediatric tracheotomy the preferred skin incision and tracheal incision, surgeon’s experience, tracheotomoy time and intubation time are important as regards development of early or late complications.

## INTRODUCTION

It is known that tracheotomoy which is thought to date back to the times before Christ has been used as a lifesaving surgical procedure for centuries. Pediatric tracheotomy (PT) is technically more difficult and risky than adult tracheotomy because of the possibility of congenital anomalies, the limited anatomical size, short and fatty neck and closeness of the vital organs.^1^,^2^

There have been some significant changes in PT surgery over the last 30 years in terms of indications. Nowadays, the extensive use of antibiotics, the development of intubation techniques, the increase in the number of patients receiving intensive care have decreased the number of tracheotomies performed due to upper respiratory tract infections such as epiglottitis and laryngotracheabronchitis and increased the number of tracheotomies done due to prolonged intubation. In PT, multifactorial causes for the development of complications include intubation time, tracheotomy experience, the preferred surgical technique of the surgeon, and the surgeon’s experience.^3^-^9^

The aim of this study was to compare the results of early and late complications which developed due to age, gender, surgical indication, intubation time, surgical experience and preferred surgical technique in children who underwent PT over the past decade in an otorhinolaryngology clinic and pediatric intensive care unit which is a tertiary reference center.

## METHODS

Children who underwent PT under elective general anesthesia in the pediatric and otorhinolaryngology clinic and pediatric intensive care unit between January 2007 and February 2017 were included in the study. The demographic characteristics of the patients, their ages, genders and indications were obtained from the files. Other information collected included indications, intubation time (0-21 days and more than 21 days), the experience of the surgeon (0-5 years and more than 5 years), surgical time (less than 30 minutes and more than 30 minutes) and surgical preferences (skin incision and tracheal incision) were compared to early and late complications.

For the statistical analysis, SPSS (Windows 22.0) was used. The Kruskal Wallis test was used for the evaluation of the relationship between the independent variables of the study and early or late complications. The findings were evaluated with a 95% confidence interval and a significance was assessed as p<0.05. The scientific ethics committee approval was taken from University of Health Sciences, Training and Research Hospital, prior to the study.

## RESULTS

Two hundred seventy three patients had PT between January 2007 and February 2017. The age distribution was between newborns and 204 months, the average age was 40.47±53.42 months (min:1, max:204) and the median was 13 months. 49.08% (134) of the children were under the age of one year; 43.2% (118) were boys and 56.8% (158) were girls.

### Indications

prolonged intubation was most common at 69.96% (191), while airway obstruction was in the second place with 30.04% (82).

### Intubation Time

the average intubation time was 17.31±8.78 days. When the intubation time was evaluated, 60.8% (166) were intubated for less than 21 days and 39.2% (107) were intubated for 21 days or more.

### Early and late complications

273 tracheotomies were performed in the children and 152 (55.68%) complications were observed. The number of complications was found to be 75 (27.47%) in the early period (in the first week) and 77 (28.21%) in the late period (after one week). Table-I, shows the distribution of complications and subgroups respectively. In our study, no significant relation was found between PT indications and early and late complications (Table-II). However while there was a significant relationship determined between children intubated for 21 days or more and the late complication, stomal-tracheal granulation (p<.000).

**Table-I:** Complications of pediatric tracheotomy.

Early and Late Tracheotomy Complications	Patients(n)	%
Early Postoperative	75/273	27.47
Bleeding	26	9.52
Accidental decannulation	21	7.69
Tube/ventilation problem	14	5.13
Subcutaneous emphysema	9	3.30
Pneumothorax	5	1.83
Late Postoperative	77/273	28.20
Tube/ventilation problem	19	6.96
Accidental decannulation	17	6.23
Stomal-tracheal granulation	12	4.40
Stomal infection	11	4.03
Bleeding	8	2,93
Subglottic stenosis	7	2.56
Tracheocutaneous fistula	3	1.10

**Table-II:** Comparison of features related to patient, surgeon and technique with early and late complications.

		Features related to patient, surgeon and technique					

		Indications	Intubation Time	Skin incision	Tracheal incision	Surgical time	Surgeon’s skill level
Early Complications	Bleeding	0.089	0.706	0.020*	0.843	0.218	0.000*
	Subcutaneous emphysema	0.574	1.000	0.954	0.025*	0.256	0.162
	Accidental decannulation	0.814	1.000	0.001*	0.074	0.000*	0.072
	Tube/ventilation problem	0.226	0.638	0.837	0.059	0.008*	0.056
	Pneumothorax	0.847	0.440	0.239	1.000	0.313	0.364
Late Complications	Bleeding	1.000	0.071	0.137	0.462	0.271	0.071
	Accidental decannulation	0.409	0.785	0.258	1.000	0.977	0.785
	Tube/ventilation problem	0.836	0.600	0.640	0.262	0.604	0.165
	Stomal infection	0.329	0.341	0.456	0.040*	0.162	1.000
	Stomal-tracheal granulation	0.282	0.000*	0.701	0.382	0.382	0.037*
	Tracheocutaneous fistula	1.000	0.082	0.243	0.243	0.596	0.082
	Subglottic stenosis	1.000	0.693	1.000	1.000	0.699	0.693

* p<0.05. (Kruskal Wallis test)

### Relation between surgery and complications

The average time of surgery was 27.84±6.49 minutes. When the surgical time and early complications were compared, there was a significant relation between the group who had a surgical time of less than 30 minutes and accidental decannulation (p<.000) and between the group who had a surgical time of more than 30 minutes and tube/ventilation problems (p<.008). It was also noted that 162 (59.3%) of the skin incisions made by surgeons were vertical and 111 (40.7%) were horizontal. When skin incision preferences and early complications were compared, a significant relation was found between horizontal incision and bleeding (p<.020), between vertical incision and accidental decannulation (p<.001). We also found that 160 (58.6%) of the tracheal incisions made by PT surgeons were vertical and 113 (41.4%) were horizontal. When tracheal incision preferences and early complications were compared, a significant relation was found between horizontal incision and subcutaneous emphysema (p<.025), and between horizontal incision and stomal infection among late complications (p<.040).

In the study, the average experience of surgeons was found to be 9.78±6.86 years. When the experience of surgeons and early complications were compared, a significant association was found between surgeons who had five years’ experience or less and bleeding (p<.000). As a late complication, a significant association was found between surgeons who had 5 years’ experience or less and stomal-tracheal granulation (p<.037).

## DISCUSSION

Two hundred seventy three PTs were performed over the past decade in our clinic for which we retrospectively evaluated the indications, intubation time, surgical techniques and surgical complication. The literature focusing on PT surgery and its complications emphasizes the age distribution of children, particularly of those under the age of one. This percentage varies between 4% and 78%.^3^-^9^ In our study, the age of the children ranged from newborn (first 6 hours) to 204 months and the average age was 40.47±53.42 months and 49.08% (134) of the children were under 12 months old. When compared to similar studies, we have a significant ratio of those under the age of one. 43.2% (118) of the 273 children were boys and 56.8% (158) were girls.^10^

Two different publications reported early complication of 19% and 22% and late complication of 51% and 77%.^9^,^11^ In our study, 273 tracheotomies were performed over the past decade and 152 (55.68%) complications were observed. The number of complications was 75 (27.47%) in the early period (within the first week) and 77 (28.23%) in the late period (after one week).

Literature review showed two major indications to be prolonged intubation or upper airway obstruction.^5^,^12^,^13^ In our study, prolonged intubation was in the first place 69.96%, and airway obstruction was in second place 30.04%. There was no significant association between the indications of tracheotomy and early and late complications in our study.

Based on today’s evidence, it is recommended to wait at least for 10-14 days to ensure that mechanical ventilation or pulmonary support is continuous before considering the need for tracheotomy.^14^,^15^ The mean intubation time in our study was 17.31 ± 8.78 days. As a late complication, there was a significant correlation between an intubation time of 21 and more days and stomal-tracheal granulation in children (p<.000). This situation may be due to the mechanical effect of the tracheotomy cannula as well as mucosal irritation and pressure associated with the cuff pressure during prolonged intubation. Perhaps it is because these children need prolonged ventilation after tracheostomy and this is probably the most likely cause.

### Surgical time

The time measured for standard tracheotomy was found to be 24.3±11.1 minutes by Oliver et al.^7^ In our study, tracheotomy surgery was performed in 27.84±6.49 minutes on average. A significant correlation was found between accidental decannulation (p<.000) and operations of 30 minutes and less; and tube/ventilation problems (p<.008) and operations of more than 30 minutes when the duration of the operation and early complications were compared. Early bleeding (p<0.218) was not found to be statistically significant; however, it was seen as a complication in 21 of 26 patients whose surgery lasted more than 30 minutes. As the duration of surgery increases, the rate of tube/ventilation problems increased which was statistically significant and incidence of bleeding increased. Interestingly accidental decannulation was higher in the group who had a surgical time of less than 30 minutes. In our opinion it is more to do with a lack of maturation sutures and poor PICU care post-op than surgical technique.

### Skin incision

The literature and our experience support the traditional use of vertical skin incision, especially in children under one year of age and in young age groups.^1^,^7^,^12^-^16^ In this study, 162 (59.3%) of the skin incisions made were vertical and 111 (40.7%) of them were horizontal. A significant correlation was found between bleeding (p<0.020) and horizontal incision and between accidental decannulation (p<0.001) and vertical incision when a comparison was made between early complications and skin incision. Incision and trachea, weakness in the supportive tissue were thought to be the cause of the significant association between accidental decannulation and vertical incision. It was thought that bleeding was more likely to occur in patients undergoing horizontal incision, due to the inability to extend the horizontal incision upward and downward, the work being carried out away from midline and the difficulties in dissection of the neck fascia and muscle groups.^1^,^7^,^12^-^14^,^16^-^18^

### Tracheal Incision

In this study, 160 (58.6%) of the tracheal incisions made were vertical and 113 (41.4%) of them were horizontal. A significant correlation was found between horizontal tracheal incision and subcutaneous emphysema (p<0.025) when the tracheal incision and early complications were compared. When late complications were observed, a significant relation was found between horizontal tracheal incision and stomal infection (p<0.040). Along with vertical tracheal incision, these also provide an advantage with maturation sutures in the placement of the cannula in the case of early decannulation. Horizontal tracheal incision is less frequently used compared to vertical incision. There are publications which indicate that horizontal incision has advantage over vertical incision in terms of tracheal collapse.^5^,^7^,^12^,^17^-^20^ Horizontal tracheal incision may cause subcutaneous emphysema. It has also been observed that horizontal incision increases the cannula pressure and the susceptibility to infection compared to vertical incision.

The experience of surgeons performing PT in our clinic was 9.78 ± 6.86 years, ranging from two years to 26 years. Bleeding (p<0.000) as an early complication and stomal-tracheal granulation as a late complication were found to be significant with surgeons with less than five years’ experience when surgeons’ experience was compared to early and late complications (p<0.037). The lack of experience of the surgeon can be a factor in bleeding complications in the early period. This situation may be associated with careless dissection or inappropriate bleeding control during surgery.^1^,^7^,^16^-^21^ Stomal granulation can be caused by inappropriate cannula selection or by pressure of the cannula to the trachea. This may be associated with a lack of experience.^16^-^23^

## CONCLUSION

Recently, the incidence of indications of prolonged intubation in PT surgery has been increasing. Determination of the need for a mechanical ventilator and the planning of tracheotomy without extending the intubation time in situations where the need is clear should be considered in order to minimize complications in terms of the surgeon’s technical preferences, surgical management and experience. Bleeding, subcutaneous emphysema and pneumothorax are complications that can be prevented by experienced surgeon accidental decannulation and tube/ventilation problem are complications that can be prevented by Intensive care and increasing the quality of clinical care services. In order to detect the tracheotomy cannula and to reduce the possibility of accidental decannulation, it is important to educate staff even though the detection implementations of tying the cannula to neck and suture on the skin were tried.
